# Is the oocyte quality affected by endometriosis? A review of the literature

**DOI:** 10.1186/s13048-017-0341-4

**Published:** 2017-07-12

**Authors:** Ana Maria Sanchez, Valeria Stella Vanni, Ludovica Bartiromo, Enrico Papaleo, Eran Zilberberg, Massimo Candiani, Raoul Orvieto, Paola Viganò

**Affiliations:** 1 Division of Genetics and Cell Biology, IRCCS Ospedale San Raffaele, Milan, Italy; 2 Obstetrics and Gynaecology Unit, IRCCS San Raffaele Scientific Institute, Milan, Italy; 3 Infertility and IVF unit, Department of Obstetrics and Gynecology, Chaim Sheba Medical Center, Tel Hashomer, affiliated to the Sackler Faculty of Medicine, Tel Aviv University, Tel Aviv, Israel; 4 Department of Obstetrics and Gynecology, Vita Salute San Raffaele University School of Medicine, IRCCS, Ospedale San Raffaele, Milan, Italy; 5 The Tarnesby-Tarnowsky Chair for Family Planning and Fertility Regulation, at the Sackler Faculty of Medicine, Tel-Aviv University, Tel Aviv, Israel

**Keywords:** Oocyte quality, Endometriosis, Follicle, Ovarian reserve, Ovary, Fertility, Granulosa cells, Cytokines, Follicolar fluid

## Abstract

Endometriosis is an estrogen-dependent chronic inflammatory condition that affects women in their reproductive period causing infertility and pelvic pain. The disease, especially at the ovarian site has been shown to have a detrimental impact on ovarian physiology. Indeed, sonographic and histologic data tend to support the idea that ovarian follicles of endometriosis patients are decreased in number and more atretic. Moreover, the local intrafollicular environment of patients affected is characterized by alterations of the granulosa cell compartment including reduced P450 aromatase expression and increased intracellular reactive oxygen species generation. However, no comprehensive evaluation of the literature addressing the effect of endometriosis on oocyte quality from both a clinical and a biological perspective has so far been conducted. Based on this systematic review of the literature, oocytes retrieved from women affected by endometriosis are more likely to fail in vitro maturation and to show altered morphology and lower cytoplasmic mitochondrial content compared to women with other causes of infertility. Results from meta-analyses addressing IVF outcomes in women affected would indicate that a reduction in the number of mature oocytes retrieved is associated with endometriosis while a reduction in fertilization rates is more likely to be associated with minimal/mild rather than with moderate/severe disease. However, evidence in this field is still far to be conclusive, especially with regards to the effects of different stages of the disease and to the impact of patients’ previous medical/surgical treatment(s).

## Introduction

The mechanisms of endometriosis-related infertility remain largely unknown [1, 2]. Several causes have been previously implicated, from anatomic distortion and tubal occlusion due to pelvic adhesions to less well-described factors such as inflammatory cytokine-mediated impairment of endometrial receptivity and oocyte quality. Surprisingly no comprehensive evaluation of the literature supporting or denying an effect of endometriosis on oocyte quality from both a clinical and a biological perspective has so far been carried out [3, 4]. This issue holds clinical relevance, as a better understanding of effect of endometriosis on ovarian function will allow better timing and tailoring of medical/surgical treatments leading to an improvement in obstetrical management of affected women. To some extent, decreased oocyte quality could in fact also be implicated in the adverse pregnancy outcomes observed in endometriosis patients [4–6]. Deeper understanding of the impact of the disease on oocyte quality also becomes fundamental as fertility preservation techniques are gaining attention in the counselling and treatment of patients affected by endometriosis [7, 8]. Both oocyte freezing and ovarian tissue cryopreservation rely on autologous folliculogenesis: disregarding any effect of endometriosis on follicular/oocyte quality at both the time of freezing and thawing may represent a major pitfall in the management of endometriosis patients.

Knowledge in this field has been hindered by the fact that most biological studies have tried to indirectly investigate the effect of endometriosis on oocyte quality, for example by studying granulosa cells (GCs) or the follicular fluid (FF) content in affected patients [9]. In the clinical setting, several indirect outcomes have been used such as the quality of the developing embryos – which is rather influenced also by the male partner [10, 11] - and clinical or live birth rates – which similarly account for several other factors including implantation and miscarriage rates.

For these reasons, we aim to present a comprehensive update on current clinical and biological data that directly relate to the effect of endometriosis on oocyte quality.

### Dysregulation of steroidogenesis

Steroidogenesis is a two-cell process, assisted by granulosa cell-derived paracrine factors that promote P450 aromatase activity - the key enzyme of the estrogen production - and allow sufficient aromatizable androgen production for an appropriate 17β-estradiol (E_2_) synthesis. E_2_ is crucial for follicular development and producing a fully competent oocyte able to reach the mature metaphase II (MII) stage and be fertilized [9]. Detrimental effects of endometriosis in the normal physiology of the GCs have been extensively described including changes in cell cycle [12], increased apoptosis [13] and dysregulation of molecular pathways involved in development and growth of GCs [14, 15]. Several studies have demonstrated that ovarian endometriosis and endometriosis at other pelvic sites may detrimentally affect GCs steroidogenesis by decreasing the expression of P450 aromatase [16].

It has previously been shown that an impairment in steroidogenesis in women with endometriosis, may lead to an imbalance in estrogen production: lower E_2_ concentrations, both at the preovulatory stage and at the luteinizing hormone (LH) surge [17]. Also, following Assisted Reproduction Techniques (ART), women affected with endometriosis seem to yield lower serum E_2_ levels on the day of hCG trigger compared with women without endometriosis [18]. Similarly, some evidence also indicates an altered postovulatory surge progesterone secretion in women with endometriosis, that might affect normal oocyte maturation [19–21].

### Dysruption of intrafollicular environment

A large number of studies have been published describing how endometriosis may alter various factors presented in the FF [16, 22–24]. The stage of the disease based on the revised classification established by the American Society for Reproductive Medicine (ASRM) [25] may have a strong importance in this context [26].

Lower FF E_2_ levels and higher FF progesterone levels in patients with endometriosis compared to controls have previously been demonstrated indicating that an impairment in steroidogenesis may directly affects the local oocyte environment [26, 27].

In recent years concerns have been raised on whether endometriosis could modify the follicular oxidative stress status. This is because oxidative stress has been proposed as being a potential factor involved in the pathophysiology of the endometriosis [28–30] and reactive oxygen species (ROS) have also been shown to promote meiotic abnormalities and chromosomic instability thereby reducing the quality of the exposed oocyte [31]. The oocyte is naturally arrested in prophase I, in which microtubule network exists as a pseudo-interphasic form. Later on, the oocyte has to form the meiotic spindle, a structure built of mostly highly dynamic microtubules [32]. The presence of a normal spindle is a pre-requisite for the acquisition of an adequate cytoplasmic and nuclear maturation, and the subsequent oocyte competence [32]. In this context, the presence of an increase oxidative stress status in the FF, has been recently confirmed in follicles surrounding an endometrioma - using proteomics by mass spectrometry [33]– and has been proposed as the culprit of spindle disruption. Accordingly, Da Broi et al. found an increase follicular 8-hydroxy-2′-deoxyguanosine, an indicator of oxidative DNA damage, in the FF of women with both mild and severe endometriosis [34, 35]. Among other factors, also brain-derived neurotrophic factor (BDNF) has been implicated as a molecular link between protection from oxidative stress and folliculogenesis [36, 37]. Specific polymorphisms in the BDNF gene have been found to be associated with incidence of endometriosis-related infertility (*p* < 0.05). Moreover, supporting the idea that a specific genotype may be implicated in infertility related to the disease, lower FF BDNF levels (*p* < 0.01), lower number of mature oocytes retrieved (*p* < 0.01) and lower fertilization rates (*p* < 0.01) were found in endometriosis patients compared to infertile patients not carrying the genotype [36]. Similarly, iron-mediated oxidative damage to the surrounding follicles has also been proposed to be associated with the presence of an ovarian endometrioma, as higher levels of iron in the FF of follicles developing adjacent to the endometriotic-cyst were observed compared to FF of follicles in contralateral healthy ovaries [38].

Nevertheless, results are still controversial: Nakagawa and colleagues failed to find an increase in the oxidative stress status in FF of women with or without endometriomas [39]. In addition, even if a recent study by Santanam et al. showed that oral supplementation with Vitamin C and E lead to a significant decrease in FF myeloperoxidase (MPO) concentration in patients with severe endometriosis undergoing IVF [40], whether oral anti-oxidant therapy might have a beneficial effect on the quality of oocytes of endometriosis patients is still far from being proved.

A damaging inflammatory milieu has also been proposed as a cause of diminished oocyte quality. Levels of cytokines in the FF and their effect on oocyte and embryo formation have indeed been extensively investigated, suggesting that cytokines, resulting both from local synthesis in the ovary and from blood/plasma, are present in the FF and might modulate folliculogenesis [24, 41]. In the context of endometriosis, altered intrafollicular levels of pro-inflammatory cytokines have been found in patients with moderate/severe disease undergoing IVF, compared to controls (with tubal factor infertility) and have also been related to the maturity of the developing oocyte: FF from follicles aspirated from endometriosis patients showed significantly higher concentrations of interleukin (IL)-8 and IL-12 compared to controls, whereas IL-8 and IL-12 levels were found to be lower in the FF of follicles containing a mature vs an immature oocyte. Thus, endometriosis-related inflammation in the FF might contribute to decreased oocyte quality [24]. Also, inflammatory ILs found at higher concentrations in peritoneal fluid of patients with endometriosis have been implicated as possible indirect disruptors to the oocyte spindle [16]. It should be noted that studies supporting the hypothesis that the peritoneal environment could indirectly influence the ovarian environment and affect spindle disruption were conducted on animal, rather than human oocytes [31, 42].

## Endometriosis and biological markers of oocyte quality

**Fig. 1:**
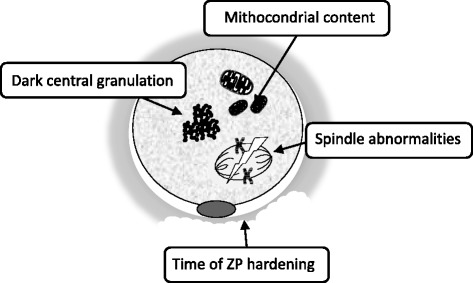
Representative morphological changes in oocytes from women affected by endometriosis

Due to ethical reasons, oocytes quality has mostly been studied indirectly through evaluation of the cumulus cells surrounding the oocytes or/and the content of the FF. However, whether these observations truly reflect the quality and the competence of the inherent oocyte is still to be elucidated [9]. For this reason, this review will summarize the existing publications analyzing human oocyte quality from a more direct point of view, such as direct observation of the oocytes (Fig. 1).

### General morphological changes

Knowledge on oocyte quality, as reflected by its morphologic and molecular characteristic, is small due to the very limited availability of MII oocytes for research. However, immature oocytes from IVF programs, that are not used in clinical treatment, can be used to study morphological changes. In fact, some publications have focused on studying the morphological changes of these immature human oocytes. Nonetheless, it should be noted that the use of oocyte morphology as an indicator of embryo quality is still uncertain. It has been hypothesized that morphological defects observed, including cytoplasmic granularity and/or the presence of vacuoles, could affect fertilization; however, the predictive value of these observations is limited due to the restrictions of non-invasive techniques as the simple transmitted light microscopy and the problem of subjectivity in the morphological evaluations. Moreover, morphology itself could be influenced by other factors, such as the ovarian stimulation or the hormonal milieu, therefore, its potential as predictive factor of clinical outcome needs further investigation [43].

In this context, Goud and colleagues performed functional studies evaluating immature oocytes derived from women with endometriosis compared to controls (women without endometriosis, regardless of their infertility factor). They found that oocytes from endometriosis women exhibited increased cortical granule loss and zona pellucida (ZP) hardening, possibly interfering with fertilization, dissolution of the ZP and the ability of embryo to undergo hatching and implantation [18] (Fig. 1.) In addition, the ability of these immature oocytes to undergo oocyte in vitro maturation (IVM) to metaphase II (MII) stage was tested, finding that a significantly lower number of germinal vesicles (GV) and metaphase I (MI) oocytes were able to reach the MII stage in the endometriosis group compared to controls [18]. A different retrospective study has evaluated the oocyte morphology in endometriosis patients. It demonstrated that the oocyte dysmorphism known as dark central granulation in the ooplasm appeared to be more frequent in oocytes from endometriosis-positive cycles [44]. More recently Borges and colleagues observed a significant increase in the incidence of extra-cytoplasmic, but not intra-cytoplasmic, oocyte defects among patients with endometriosis. Endometriosis was negatively correlated with embryo developmental potential, while the blastocyst formation rate remained unaltered. No information about blastocyt quality was provided in this study [45].

### Spindle abnormalities

There are over 100 proteins associated with the spindle apparatus and its movement, but its main structural component is a dimer consisting of α- and β-tubulin subunits. In normal conditions, the spindle apparatus of an oocyte should be compact, made up of discrete spindles, with nothing visible between them. As this structure must coordinate the alignment and normal segregation of homologous chromosomes and sister chromatids in the two subsequent meiotic divisions, disruption of the meiotic spindle results in abnormal chromosome alignment and fertilization. In the context of ICSI cycles, the outcomes of oocytes with normal spindles have been found superior to those of oocytes without normal spindles in terms of fertilization rates [46], and also with regard to euploidy rates of the resulting embryos [47]. In recent years, spindle morphology has emerged as a marker of oocyte quality and hence many technological advances have been made to visualize this structure. Two of these methods are confocal microscopy and polarized light microscopy. Confocal microscopy, however, requires the sample to be fixed and stained, so it is not an option for oocytes intended for use in IVF/ICSI. Polarized light overcomes this limitation and makes the real-time visualization of the spindle possible [48]. Since advancing maternal age is a well-recognized factor influencing the occurrence of spindle abnormalities, maternal systemic/local conditions non related to aging might also play a role [48]. In a pilot study, Barcelos and colleagues (2009) analyzed oocyte spindle morphology after IVM, comparing oocytes from endometriosis patients (*n* = 35) with oocytes from a control group with tubal factor infertility (*n* = 19). Both IVM rates and the incidence of meiotic abnormalities such a spindle disruption or chromosomal misalignment were not statistically different between the two groups. However, the authors observed that the number of oocytes studied was too low to detect statistically significant differences [49]. In a similar study conducted in the context of IVM, Dib and colleagues did not find any differences in the number of matured oocytes with visible spindles and in spindle location, among endometriosis patients and controls with male/unexplained infertility. However, a significant decrease in the number of fertilized oocytes was found in patients with moderate/severe endometriosis but not with minimal/mild disease [50].

In contrast, a more recent study by Goud and colleagues, using the same technique of IVM, found a higher percentage of abnormal spindles in oocytes retrieved from women with endometriosis compared to women undergoing ART due to male factor (66.7% vs 16%, *p* < 0.05) [18]. Finally, in the setting of conventional ICSI, Rajani et al. (2012) compared the oocytes spindle morphology in patients with endometriosis, polycystic ovarian syndrome (PCOS) and tubal factor infertility (controls), and found that PCOS, but not endometriosis, was characterized by lower spindle visibility compared to controls [51].

It should be considered that studies addressing the spindle morphology to assess oocyte quality in these patients have evaluated oocytes from IVM protocols that could not reflect the mature oocyte spindle configuration [52]. The only study that observed the spindle in mature oocytes was the one from Rajani et al. [51] that failed to find significant alterations in endometriosis patients. In conclusion, the visualization or not of the meiotic spindle as a marker of oocyte quality in women with endometriosis needs further investigation.

### Cytoplasmic ultrastructure: Low mitochondrial content

Cytoplasm composition is crucial in oocyte competence and embryo development [53]. In particular, cytoplasm of mature oocytes has a very high mitochondrial content compared to other cell types, as it can contain up to 10^5^ mitochondria [54–56]. It is generally accepted that mitochondrial abnormalities and/or dysfunction – as observed in association with maternal ageing - could have an adverse influence on fertilization of human oocytes and human embryonic development [53, 57–60]. Surprisingly, the effect of endometriosis on oocyte cytoplasm morphology has, however, received limited attention.

To our knowledge, the first and only study that has evaluated the association between the cytoplasm ultrastructure of oocytes and the presence of endometriosis has been done by Xu et al. in the context of ICSI cycles. In this study a total of *n* = 50 MII oocytes from patients with laparoscopically diagnosed minimal/mild endometriosis and control women (with tubal or male factor infertility), was studied by means of transmission electron microscopy (TEM). They showed that a higher number of nuclei of the oocytes from women with endometriosis contained decentralized chromatin and a voluminous nucleolus compared to those from control group. Moreover, oocytes from women with endometriosis had both a higher percentage of abnormal mitochondria (containing small or swollen and blurred vacuoles) and an overall lower number of mitochondria. This observation was also confirmed by a lower number of mitochondrial DNA (mtDNA) copies found with quantitative real time PCR in oocytes from women with endometriosis compared with controls (84,6 ± 39,8 vs 50,7 ± 288,5, Mean ± SD, *p* < 0.05). No differences were instead found in the morphology of the cortical granules, Golgi apparatus and spindles between both groups. They concluded that low mtDNA content specifically reflects decreased oocyte quality in women with minimal or mild endometriosis. Unfortunately, no clinical data were reported in this study [4].

## Endometriosis and clinical markers of oocyte quality

A significant number of women with endometriosis eventually seek IVF to achieve pregnancy and the effect of this pathology on IVF outcomes has been studied extensively. Several meta-analysis studies have reported on the effect of the endometriosis on IVF outcomes as pregnancy rates, miscarriage rates or live birth rates, but only few of them evaluated the effect of the endometriosis on parameters directly correlating with oocyte quality. As oocyte competence is well defined as the ability of the oocyte to complete maturation and undergo successful fertilization [61], the condition of poor oocyte quality might be clinically represented by a lower number of MII oocytes retrieved and a lower fertilization rate.

Aiming to comprehensively update the clinical data regarding the quality of the oocytes in patients affected by endometriosis, we have systematically retrieved meta-analyses describing data on number of MII oocytes and fertilization rates in IVF/ICSI cycles of women affected by endometriosis. Relevant clinical studies conducted after the publication of the last meta-analysis were also individually retrieved. In addition, studies from oocyte donation experiences of patients with endometriosis will be also discussed.

### Meta-analysis studies and recent evidence

Four meta-analyses provided clinical insights on the effect of endometriosis on oocyte quality (Table 1). The first, published by Barnhart and colleagues (2002), compared the IVF outcome of women with endometriosis, taking into account the different stages of the disease, with women with other causes of infertility (tubal factor, male factor, ovulatory dysfunction). Overall, the authors included data from 22 non-randomized studies for a total of *n* = 2377 IVF cycles of women with endometriosis and *n* = 4383 IVF cycles from non-affected women, even if the number of studies included in each sub-analysis was not reported. Their adjusted analysis reported a lower fertilization rate in endometriosis patients [odds ratio (OR), 0.81; 95% confidence interval (CI), 0.79–0.83, *p* < 0.001)] supporting a deleterious impact on oocyte quality. Then, they separately compared women with minimal/mild disease and those with moderate/severe disease [25], with women with tubal factor infertility. The fertilization rate in women with severe endometriosis was higher than that in women with tubal factor infertility (OR 1.54; 95%CI, 1.39–1.70, *p* < 0.001), or women with minimal/mild endometriosis (OR 1.11; 95%CI, 1.09–1.13, *p* < 0.001) [62]. Unfortunately this meta-analysis did not distinguish between women who received previous medical and/or surgical treatments, thus potentially weakening the associations identified [62].

**Table 1:** Results from meta-analysis studies providing clinical insights on the effect of endometriosis disease on oocyte quality.

First author, Year [Ref]	Endometriosis groups^a^	Outcome: MII oocytes retrieved		Outcome: Fertilization rate	
		Studies included (n)	Endometriosis vs controls (95% CI)	Studies included (n)	Endometriosis vs controls (95% CI)
Barnhart, 2002 [56]	Overall Stage I-II Stage III-IV			Unknown Unknown Unknown	OR 0.81 (0.79 to 0.83)^c^ OR 0.94 (0.93 to 0.96)^c^ OR 1.54 (1.39 to 1.70)^c^
Harb, 2013 [57]	Untreated Stage I-II Untreated Stage III-IV			7 3	RR 0.93 (0.87 to 0.99)^c^ RR 1.01 (0.93 to 1.10)
Yang, 2015 [58]	Untreated endometrioma	2	MD −3.61 (−4.44 to −2.78)^c^	2	OR 1.06 (0.71 to 1.60)^b^
Rossi, 2016 [59]	Overall Stage I-II Stage III-IV Treated disease (surgery) Untreated disease Endometrioma	4 2 3 3 1 2	OR −1.22 (−2.38 to −0.06)^c^ OR −0.55 (−1.34 to 0.25) OR −0.83 (−1.73 to 0.08) OR −1.62 (−3.31 to 0.07) OR −0.50 (−1.59 to 0.59) OR −2.48 (−4.43 to −0.53) ^c^		

*MD* mean difference, *OR* odds ratio, *RR* relative risk

^a^Where not specified, history of treatment vs untreated disease is unknown

^b^Control group: contralateral healthy ovary

^c^Statistically significant

Another and more recent meta-analysis from Harb and colleagues (2013), where studies of women who had received medical or surgical treatment for endometriosis before the IVF cycles were excluded, found a 7% reduction in fertilization rate from seven studies including only minimal/mild endometriosis [relative risk (RR) 0.93, 95%CI 0.87–0.99*, p* = 0.03]. However, this effect was small, mainly due to the low number of studies included (*n* = 7) and the significant heterogeneity between them - as indicated by an I^2^ value of 66% (*p* = 0.008). In contrast, no significant reduction in fertilization rates was found for moderate/severe disease compared to controls (RR 1.01, 95%CI 0.93–1.10, *p* = 0.84) even if this sub-analysis was only based on three, heterogeneous studies (I^2^ value = 70%, *p* = 0.03) [63].

An independent and crucial discussion remains, whether the presence of a non-surgically treated ovarian endometrioma alone may adversely affect the oocyte quality. In this case, only one meta-analysis including only 9 studies has been published [64]. In this review, Yang and collaborators reported that the total number of oocytes retrieved were 1.5 fewer in women with ovarian endometrioma, compared to those without [weighted mean difference (WMD) -1.5; 95%CI -2.84 to −0.15, *p* = 0.03)]. Moreover, the number of MII oocytes retrieved in the ovarian endometrioma group was 3.61 fewer (WMD - 3.61; 95%CI -4.44 to −2.78, *p* < 0.000001). In terms of fertilization competence, only 2 studies that compared the fertilization rates between the ovary with endometrioma and the contra-lateral healthy ovary were included in the meta-analysis, showing no difference (OR 1.06, 95% CI 0.71 to 1.60, *p* = 0.77) [64]. The main limitations of this meta-analysis are the clinical heterogeneity of the studies and the low sample size.

Another recent meta-analysis reported a decreased number of mature oocytes retrieved in women with endometriosis (treated/untreated) compared to women with other causes of infertility (OR -1.22, 95%CI -2.38 to −0.06). Again the total number of retrieved oocytes was lower (OR -1.93, 95%CI -3.67 to −0.18) and fertilization rates were not included in the analysis. It is thus difficult to determine whether this result should be related to a qualitative or rather a quantitative impairment in ovarian reserve, possibly also due to previous surgical treatments. When providing subgroup analyses restricted to different stages of endometriosis or previous treatments (See Table 1), the same authors did not find any significant effect on number of mature oocytes retrieved compared to controls except for patients with ovarian endometrioma (OR -2.48, 95% CI 4.43 to −0.53). Unfortunately, whether any treatment was received for the ovarian endometrioma was not specified [65]. In addition, only one to three studies were included in the above-mentioned sub-analyses and the retrieval of the specific included studies included is not possible, hindering further interpretation of these observations [65].

Other recent meta-analyses on the outcomes of ART in patients with endometriosis unfortunately do not provide data on the quality of oocytes as indicated by number of MII oocytes retrieved or fertilized [66, 67]. For this reason, recent publications providing additional evidence were individually retrieved and are herein discussed.

Three retrospective studies have compared women with ovarian endometriosis with controls with tubal factor infertility. In two of the studies including patients who were surgically treated for moderate/severe endometriosis, a slight reduction in fertilization rate was observed compared to controls [61.6% vs 64.0%, *p* = 0.03 and 64.8% vs 70.2%, *p* = 0.04 in the study by Dong et al. (2013) and Singh et al. (2014), respectively] [68, 69]. In a third, very small study by Luca et al. including patients with known ovarian endometriosis and without previous surgery, no differences in number of MII oocytes retrieved were reported compared to controls. However, sample size calculation was lacking and a lack of statistical power is very likely [70]. Harada and colleagues compared the outcomes of IVF in the ovary following an excision of endometrioma with the contralateral ovary, demonstrating a lower number of oocytes retrieved (*p* = 0.009) with comparable fertilization rates. Again, sample size calculation analysis was not reported [71]. Results from two other small studies by Pop-Trajkovic and colleagues, reporting similar fertilization rates for minimal/mild endometriosis and controls, with higher fertilization rates in moderate/severe disease, are also difficult to interpret because the fertilization rates were unexpectedly as low as 54% for controls with tubal factor infertility, and again sample size calculation was not available [72, 73].

More recently, two prospective case-control studies found similar results and confirmed significantly lower number of mature oocytes and lower fertilization rates in patients with endometriosis compared to patients with other causes of infertility [24, 74]. In both studies, unfortunately, no information regarding previous surgical treatments received is provided. In particular, in the study by Shebl and colleagues, all stages of endometriosis were included and mature MII oocytes were also screened for morphological anomalies (vacuoles, refractile bodies, perivitelline space anomalies, aggregation of the smooth endoplasmic reticulum, central granulation, brownish discoloration, and ovoid shape). Fewer morphologically normal oocytes were observed in endometriosis patients compared to controls (*p* < 0.001) and fertilization was significantly reduced in endometriosis patients compared to controls in conventional IVF (44.9% vs 54.4% respectively, *p* < 0.03) but not in ICSI (74.9% vs 76.9% respectively, *p* = 0.38). In addition, severe endometriosis was associated with significantly worse-quality oocytes than less severe stages (*p* < 0.01) [74].

Lastly, a large retrospective study based on the register from the American Society of Reproductive Technology, was also recently carried out [1]. The study compared the outcomes of *n* = 33458 cycles where endometriosis was present either alone (*n* = 12335) or with other concomitant infertility-related diagnoses (*n* = 21123) to cycles due to tubal factor infertility (*n* = 22778), unexplained infertility (*n* = 38713) and other causes of infertility (*n* = 196295). Among the results, we deem relevant with respect to the present review that a significant reduction in fertilization rate compared to women with tubal factor infertility was found both in women with endometriosis only (RR 0.97, 95% CI 0.96 to 0.98, *p* = 0.0001) and in women with endometriosis and concomitant diagnoses (RR 0.98, 95% CI 0.97 to 0.99, *p* = 0.0001) [1]. However, the retrospective, registry-based nature of the data, as well as the absence of information about previous endometriosis treatment(s) represent reasons for caution in the interpretation of the results.

### Oocyte donation experiences

Oocyte donation programs have also been studied in the past to provide interesting clinical insights into the main causes of endometriosis related-infertility [75]. One of the first studies that tried to gain clinical knowledge of the factors involved in the aetiology of the endometriosis-associated infertility was the study by Simon and colleagues [76]. They demonstrated that patients with endometriosis have the same chances of implantation and pregnancy as other recipients when the oocytes came from healthy donors. In contrast, patients who received embryos derived from endometriotic ovaries showed a significantly reduced implantation rate as compared to the remaining groups (*p* < 0.05) and hypothesized that this observation was related to oocyte quality.

Later, the same group published a prospective study, in which three groups were established in order to eliminate the inherent bias of a retrospective nature: group 1, contained donors and recipients without endometriosis (*n* = 44); group 2, donors with endometriosis that provided oocytes to recipients without (*n* = 14) and group 3 donors and recipients with endometriosis (*n* = 16). The impairment of pregnancy rate per transfer observed in group 2 confirmed that the embryos derived from oocytes from women with endometriosis may have lower ability to implant due to alterations within the oocyte [77].

The fact that endometrial receptivity is spared in oocyte donation recipients affected by endometriosis, was later confirmed by Sung et al. (1997) in a retrospective analysis on 239 consecutive oocyte recipients (patients with endometriosis *n* = 55, patient without *n* = 184), where no difference in pregnancy rates between the two groups was found [78].

Furthermore, in a prospective matched case-control study by Diaz et al.*,* 2000, IVF outcomes of women with or without endometriosis that received ‘siblings’ oocytes from the same “healthy” donor were evaluated in an attempt to avoid the bias of assigning oocytes of different quality to the different groups. Pregnancy, implantation, and miscarriage rates were not affected by moderate/severe endometriosis when compared with the control group [79].

Overall, all this studies support the idea that infertility of endometriosis patients could be not related to endometrial environment but rather to a diminished oocyte quality. More recently, a small retrospective study by Katsoff et al. did not find any differences in clinical pregnancy and live birth rates among recipients receiving eggs from donors with (*n* = 21) or without (*n* = 133) laparoscopically diagnosed endometriosis. However, power calculation analysis was lacking and results are thus difficult to interpret [80].

## Potential impact on clinical practice

Several relevant questions that may impact the clinical practice remain largely unanswered. For example, the extent to which the oocyte quality observed in patients with endometriosis may be influenced by previous medical/surgical treatment(s) is unfortunately still difficult to discern [81]. In fact, only one existing meta-analysis included only studies where patients had not received any medical/surgical treatment(s) before ART [63]. While a decrease in fertilization rates for minimal/mild endometriosis was confirmed compared to other causes of infertility, unfortunately the effect of endometriosis per se on the number of mature oocytes retrieved was not assessed as in other meta-analyses in the field [67]. Another meta-analysis that specifically focused on the presence of ovarian endometrioma at the time of ART, included only patients who had not received any previous medical/surgical treatment(s). Here, the authors found a significant decrease in the number of MII oocytes retrieved when an ovarian endometrioma was present compared with patients without endometriosis, but also an overall lower number of oocytes collected were reported. Thus this observation might also be due to a lower oocyte yield due to the presence of the cyst at the time of the oocyte retrieval, rather than to a qualitative damage to the oocytes [64]. However, an interesting recent study including ovarian cortex biopsies from ovaries with endometrioma and contralateral ovaries derived from 13 women <40 years of age, showed a significant increase in oocyte apoptosis and follicle atresia in the affected ovary. Importantly, women included in the study had “small” endometriomas (range 1–4 cm, median 2.7 cm) and thus a negative effect for early stages per se seems to be confirmed [82].

On the other hand, endometriosis is a heterogeneous disease with different stages and clinical phenotypes, and their influence on oocyte quality requires further studies. In this context, clinical strategies to prevent or minimize the detrimental effect of endometriosis on oocyte quality should be considered and an effort to identify subgroups of patients who may benefit from treatments should be done. Surgical treatment of endometriosis is very unlikely an option when aiming at improving oocyte quality, due to the well-known impact that surgery has on ovarian reserve [83]. In contrast, medical therapies should be explored, as they could become relevant pre-medications in the context of fertility treatments.

The use of a 3–6 months of GnRH agonist treatment prior to IVF/ICSI in patients with endometriosis has been previously suggested to improve clinical pregnancy rates, but whether this effect was to be related to an endometrial or an ovarian benefit remained unexplained, according to the results of the Cochrane review by Sallam et al. (2006) [3]. More recently, a randomized controlled trial (RCT) tested the efficacy of a 3-month-long GnRH agonist treatment prior to IVF, looking at the number of MII oocytes retrieved as the main outcome, and failed to find any benefit. Predictably, higher doses of FSH and a longer stimulation were also required for ovarian stimulation in the group receiving GnRH agonist pre-treatment. However, patients included were women not affected by ovarian endometriosis and surgically treated for peritoneal endometriosis, who were either assigned to immediate IVF after surgery or to post-surgical 3-month GnRH agonist treatment and subsequent IVF [84]. Hence, no information can be obtained about the benefits of GnRH agonist in the presence of ovarian endometriomas or in patients who have not undergone surgical treatment for endometriosis. Further RCTs are therefore required.

In addition, based on current evidence, ICSI should be preferred over conventional IVF in patients with endometriosis. In fact, a previous prospective randomized study on patients with laparoscopically diagnosed moderate/severe endometriosis and normozoospermic partners, including a total of *n* = 786 oocytes showed that, sibling oocytes achieved significantly higher fertilization rates with ICSI rather than conventional IVF (73.3% vs 54.7%, *p* < 0.01), suggesting that ICSI might be preferable over conventional IVF in endometriosis-associated infertility [85].

Current literature, unfortunately, does not provide any insight into the survival rates of frozen/thawed oocytes from women with endometriosis compared to controls without the disease. Hence, the extent to which the negative effect of endometriosis on oocyte quality might also impair the performances of fertility preservation techniques remains unexplored and this option should still be considered with caution [7].

## Conclusions

Based on the review of the current literature, endometriosis seems to negatively affect oocyte quality, in terms of several relevant clinical and biological outcomes. As oocyte quality is well reflected by the ability to complete maturation and undergo successful fertilization, the best clinical markers of oocyte competence are represented by number of MII (mature) oocytes retrieved and fertilization rates observed in IVF/ICSI outcomes. The hitherto published available evidence seems to suggest that a reduction in the number of mature oocytes retrieved is consistently associated with endometriosis, compared to other causes of infertility [22, 65–67, 74], while a reduction in fertilization rates is likely to be associated more with minimal/mild rather than with moderate/severe endometriosis [62, 72, 73].

In the context of biological studies, oocyte quality has instead been investigated by means of morphological assessment, and by the ability to undergo IVM, spindle imaging or by cytoplasmic ultrastructure imaging. The available evidence tends to prove that oocytes retrieved from women affected by endometriosis are more likely to fail IVM and to show altered morphology and lower cytoplasmic mitochondrial content compared to women with other causes of infertility.

All the aforementioned observations may support the notion that infertility of endometriosis patients should be related to diminished oocyte quality, rather than endometrial environment/receptivity. On the other hand, these observations are still controversial and some unresolved aspects should be considered: first, conflicting results have been reported for all the above-mentioned outcomes. Different studies have failed to find a significant impairment in oocyte quality between patients with endometriosis and controls, especially in the context of biological investigations based on spindle visualization [18, 46–51]; secondly, a deeper look at these studies reveals that most of them lack the statistical power to exclude an effect of endometriosis on oocyte quality, due to insufficient sample size.

In conclusion, to our knowledge this is the first review to summarize the results of both clinical and biological studies available on the impact of endometriosis on oocyte quality. We found that a detrimental effect of endometriosis on oocyte quality seems to be present. Based on the aforementioned studies - in patients with endometriosis undergoing ART - ICSI should likely be preferred over conventional IVF, and the role for a 3–6 months GnRH agonist pre-treatment deserves further research. However, evidence in this field is still far to be conclusive, especially with regards to the effects of different stages of endometriosis and previous treatments received on oocyte quality.

## Abbreviations

ART: Assisted Reproduction Techniques
BDNF: brain-derived neurotrophic factor
E_2_: Estradiol
FF: Follicular Fluid
GC: Granulosa Cells
IVM: oocyte in vitro maturation
MPO: myeloperoxidase
mtDNA: lower number of mitochondrial DNA
PCOS: Polycystic Ovarian Syndrome
ROS: reactive oxygen species
TEM: transmission electron microscopy
ZP: zona pellucida
